# Battle at the entrance gate: CIITA as a weapon to prevent the internalization of SARS-CoV-2 and Ebola viruses

**DOI:** 10.1038/s41392-020-00405-2

**Published:** 2020-11-24

**Authors:** Rafal Butowt, Krzysztof Pyrc, Christopher S. von Bartheld

**Affiliations:** 1 L. Rydygier Collegium Medicum, Nicolaus Copernicus University, Bydgoszcz, Poland; 2 Virogenetics Laboratory of Virology, Malopolska Centre of Biotechnology, Jagiellonian University, Gronostajowa 7a, 30-387 Krakow, Poland; 3 Center of Biomedical Research Excellence in Cell Biology, University of Nevada, Reno School of Medicine, Reno, NV USA

**Keywords:** Infectious diseases, Infectious diseases, Diseases of the nervous system

In a recent paper in *Science*, Bruchez et al.^1^ provide new insights how host cells can fight virus infection. They show that an MHC class II transactivator (CIITA)—which normally operates as part of the interferon (IFN)-stimulated immune response—is also involved in an antiviral response, beyond its well-known function of antigen presentation.

**Fig. 1:**
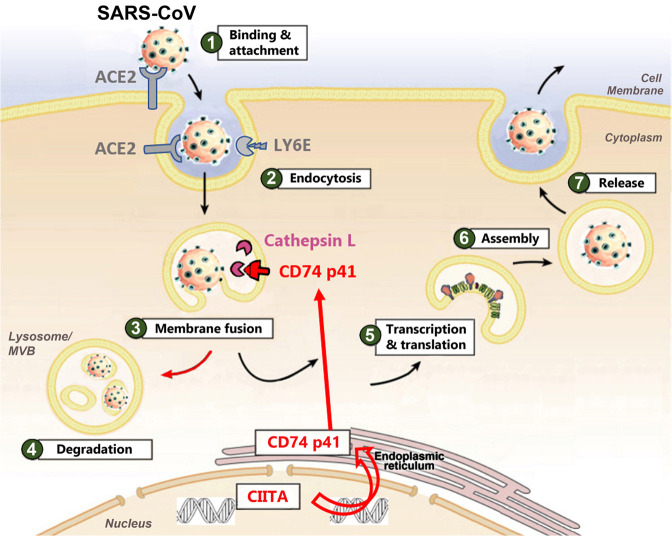
Interference of coronavirus invasion by preventing fusion of viral and host membranes in the endosomal pathway. The coronavirus (SARS-CoV) enters host cells by binding to ACE2 (1), followed by endocytosis (2) and cathepsin-mediated cleavage of the viral glycoproteins. Glycoprotein cleavage allows fusion of viral membranes with endosomal membranes (3) and release of viral RNA into the cytoplasm. CIITA (class II major histocompatibility complex transactivator) upregulates the CD74p41 isoform, which inhibits cathepsins and prevents genome release into the cytoplasm, instead redirecting the virus into a degradation pathway in multivesicular bodies (MVBs) or lysosomes (4). This mechanism aborts or reduces viral transcription/translation, assembly, and virus release (5–7, right side)

Antiviral mechanisms adopted by host cells to avoid viral infection are well studied, but some gaps remain. This is especially applicable for coronaviruses (CoVs), which were understudied for years. Cells have evolved multiple antiviral strategies to impede the virus at early steps of viral infection, many of them dependent on IFN stimulation. The host cell attempts to limit the number of viruses that enter the cells. One of the strategies aims to reduce viral invasion by expression of the GPI-anchored LY6E protein, which can inhibit the fusion of viral and host membranes (Fig. 1).^2^ Another novel mechanism was recently discovered by Bruchez et al.^1^

The authors utilized U2OS cells, a human osteosarcoma cell line, and performed much of the work on Ebola virus, as already summarized.^3^ In this research highlight, we emphasize the implications for SARS-CoV-2, also examined by Bruchez et al., because of the current health threat associated with this virus. The authors used a transposon-based screening approach to identify which activated or inactivated genes affect virus infection and then established that the CIITA transcription factor inhibited virus processing. Further analysis revealed that CIITA activates transcription of CD74 protein isoform p41, which is delivered to the endosomal pathway (Fig. 1). This response prevents the priming of the surface glycoproteins of Ebola virus and CoVs by a particular class of cathepsins. The role of cathepsins during coronaviral infection of the human respiratory tract is minimal, but these endosomal enzymes may play an essential role during systemic infection in other tissues. The CD74p41 isoform contains a thyroglobulin domain, which is responsible for blocking cathepsin L, which, in turn, prevents viral spike glycoprotein proteolytic priming and activation. This blocks viral membrane fusion to the endosomal host membrane and thereby prevents the release of viral RNA to the cytoplasm. While the role of cathepsins in the priming and activation of several viruses including Ebola and CoVs has been shown previously,^4^ Bruchez et al. now reveal a novel role for CIITA and CD74p41, in an IFN-dependent antiviral response at the early stage of viral infection. These new findings provide a mechanistic account that suggests therapeutic strategies to block the COVID-19 infection and further confirms that among proteases in the endosomal compartment, cathepsins are promising targets. The findings by Bruchez et al. also suggest that genetic manipulation and/or novel drugs manipulating the levels of CIITA and CD74p41 could be more effective than direct chemical inhibitors of cathepsin such as teicoplanin derivatives, but with less toxicity.

Bruchez et al. report the accumulation of the pseudotyped Ebola virus in multivesicular bodies (MVBs) in vitro. MVBs are intermediate endosomal compartments, which may fuse with lysosomes for degradation but may also release exosomes. This suggests that pseudotyped Ebola virus in MVBs, and possibly also SARS-CoV-2, may be released to the extracellular environment. Thus, MVBs may contribute to the intercellular transfer of SARS-CoV-2 independent of angiotensin convertase enzyme 2 (ACE2), the SARS-CoV entry protein (Fig. 1). This may have important implications for some tissues, including the brain, where low ACE2 levels are present, but intercellular virus transfer was documented postmortem. Whether the action of cathepsins on CoVs and Ebola virus surface glycoproteins occurs in late endosomes, in MVBs, or exclusively in lysosomes requires further investigation. Cathepsins were shown to be present not only in lysosomes but also in late endosomes, and the activation of viral spike protein by cathepsins likely takes place in this compartment (Fig. 1).

But how can therapies be implemented when the virus has already infected cells and is replicating? The brain typically shows delayed neurological symptoms because it is not an immediate target of SARS-CoV-2 infection. The findings of Bruchez et al. may be particularly important for brain infection where microglia, the brain’s innate immune cells (similar to peripheral blood monocytes and macrophages), express the ACE2 SARS-CoV-2 receptors as well as the CIITA transcription factor, according to large-scale transcriptomic data. Some macrophages in the periphery, e.g., in the alveoli, are infected by SARS-CoV-2, and this contributes to the lung damage. Proinflammatory IFN/cytokine stimulation may increase levels of CIITA in the microglia, thereby protecting these cells from infection with SARS-CoV-2. Once microglial cells become infected, immediate neuronal damage can be expected, emphasizing the need for protection of these cells. Moreover, TMPRSS2 protease, which was shown to be the key player in the lungs and is the main SARS-CoV-2 spike protein priming enzyme, is not or only minimally expressed in the adult brain. It remains to be determined whether other cell surface proteases are able to carry out this process—triggering the virus fusion on the cell surface, or whether the cathepsin levels in the brain are sufficient to mediate endosomal entry. Depending on the outcome, therapeutic strategies aimed to increase CIITA and/or CD74p41 may be particularly suitable to reduce brain damage and to prevent long-term neurological symptoms observed in COVID-19.

We must emphasize that any therapeutic strategy designed for the current COVID-19 pandemic may have side effects. Some antiviral strategies to inhibit viral entry based on LY6E protein (Fig. 1) may protect from one group of viruses such as CoVs and Ebola virus, but may facilitate cell entrance and infection by other virus types such as flaviviruses or influenza A virus.^2^ This potential double-edged nature of antiviral therapies must be considered in future approaches targeting the CIITA/CD74p41 pathway. Furthermore, antiviral effects need to be confirmed in additional cell lines in vitro, and many antivirals have failed to be effective in human trials. Mutational changes in the SARS-CoV-2 genome, such as the D614G alteration, may reflect an adaptive virus response to these cellular mechanisms. It was shown in recent studies that the D614G mutation in the spike protein increases the efficiency of SARS-CoV-2 cell entry and infection rate in vitro and in vivo,^5^ which may counteract the above described cellular strategies.
